# Is intraoperative ultrasound more efficient than magnetic resonance in neurosurgical oncology? An exploratory cost-effectiveness analysis

**DOI:** 10.3389/fonc.2022.1016264

**Published:** 2022-10-28

**Authors:** Alejandra Mosteiro, Alberto Di Somma, Pedro Roldán Ramos, Abel Ferrés, Andrea De Rosa, Sofía González-Ortiz, Joaquim Enseñat, Jose Juan González

**Affiliations:** ^1^ Department of Neurosurgery, Hospital Clínic de Barcelona, Barcelona, Spain; ^2^ Facultad de Medicina, Universitat de Barcelona, Barcelona, Spain; ^3^ Division of Neurosurgery, Università degli Studi di Napoli “Federico II”, Naples, Italy; ^4^ Department of Radiology, Hospital Clínic de Barcelona, Barcelona, Spain

**Keywords:** intraoperative magnetic resonance, intraoperative ultrasound, neurosurgical oncology, glioma, cost-effectiveness

## Abstract

**Objective:**

Intraoperative imaging is a chief asset in neurosurgical oncology, it improves the extent of resection and postoperative outcomes. Imaging devices have evolved considerably, in particular ultrasound (iUS) and magnetic resonance (iMR). Although iUS is regarded as a more economically convenient and yet effective asset, no formal comparison between the efficiency of iUS and iMR in neurosurgical oncology has been performed.

**Methods:**

A cost-effectiveness analysis comparing two single-center prospectively collected surgical cohorts, classified according to the intraoperative imaging used. iMR (2013-2016) and iUS (2021-2022) groups comprised low- and high-grade gliomas, with a maximal safe resection intention. Units of health gain were gross total resection and equal or increased Karnofsky performance status. Surgical and health costs were considered for analysis. The incremental cost-effectiveness ratio (ICER) was calculated for the two intervention alternatives. The cost-utility graphic and the evolution of surgical duration with the gained experience were also analyzed.

**Results:**

50 patients followed an iMR-assisted operation, while 17 underwent an iUS-guided surgery. Gross total resection was achieved in 70% with iMR and in 60% with iUS. Median postoperative Karnofsky was similar in both group (KPS 90). Health costs were € 3,220 higher with iMR, and so were surgical-related costs (€ 1,976 higher). The ICER was € 322 per complete resection obtained with iMR, and € 644 per KPS gained or maintained with iMR. When only surgical-related costs were analyzed, ICER was € 198 per complete resection with iMR and € 395 per KPS gained or maintained.

**Conclusion:**

This is an unprecedented but preliminary cost-effectiveness analysis of the two most common intraoperative imaging devices in neurosurgical oncology. iMR, although being costlier and time-consuming, seems cost-effective in terms of complete resection rates and postoperative performance status. However, the differences between both techniques are small. Possibly, iMR and iUS are complementary aids during the resection: iUS real-time images assist while advancing towards the tumor limits, informing about the distance to relevant landmarks and correcting neuronavigation inaccuracy due to brain shift. Yet, at the end of resection, it is the iMR that reliably corroborates whether residual tumor remains.

## Introduction

Intraoperative imaging is a major asset in modern neurosurgical oncology which helps the surgeon delineating tumor boundaries and identifying remnants (1–3). It ultimately improves the extent of resection (EoR), a major prognostic factor in both high (4, 5) and low-grade gliomas (6, 7), as well as in brain metastasis (8).

Imaging devices have evolved during the last decades, becoming more precise, versatile and accessible. Still, each modality has its own strengths and shortcomings (3). Intraoperative ultrasound (iUS) is convenient in terms of costs, maneuverability and it provides real-time representations of the operative field. However, it is an operator dependent technique and it has restricted resolution for tissue differentiation (9). In contrast, intraoperative magnetic resonance (iMR) is considered the prime study for brain assessment, with high accuracy in tissue definition and reliable for achieving gross total resection of brain tumors (10, 11). But iMR requires specific infrastructure and high initial investment (12). Moreover, its long acquisition times and the particular workflow required increase the operative duration.

Following the differential features of intraoperative imaging devices, tertiary neurosurgical centers have been choosing between modalities according to their preferences and prospects. However, a formal comparison of the efficiency between iUS and iMR in the neurosurgical oncology setting has not been performed yet. Hereby, we have evaluated the cost-effectiveness of iUS and iMR for brain tumor resection. Comparing the economic costs and health benefits of these two alternative interventions will provide objective data for decision makers and future investments.

## Methods

### Population of reference

The patients included in this retrospective analysis were part of two prospectively collected clinical registries. One cohort was composed of consecutive patients treated from high and low grade gliomas, with a maximal safe resection intention, with the assistance of a low field-iMR (PoleStar N-20, Odin Medical Technologies, Yokneam, Israel and Medtronic, Louisville, CO, USA). These patients were recruited between June 2013, date of the installation of the device, and June 2016. The data corresponding to this cohort has already been published in this same journal by our group (13).

The second cohort consisted of patients treated for high- and low-grade gliomas, with a maximal safe resection intention, with the aid of an iUS (bk5000 neurosurgical system, BK Medical, Burlington, Massachusetts, USA) and a specific neurosurgical probe (bk Craniotomy Transducer N13C5). No other iUS appliances were used, neither 3D reconstructions nor co-registration with the neuronavigation system. Intraoperative contrast agents were not applied. These patients were operated between October 2021, date of acquisition of the device, and May 2022.

Neurophysiologic monitoring was implemented in both cohorts, whenever the surgical team considered it appropriate. In cases with initial suspicion of high-grade glioma, intraoperative fluoresce with 5-aminolevulinic acid (Gliolan ^®^) was additionally used to guide the resection. In all the cases, neuronavigation was employed to tailor the craniotomy and to aid with the resection. Patients in which both intraoperative devices were used were excluded from the analysis; they belong to the intermediate time period (2016-2021).

The study research was approved by the institutional review board (HCB/2013/8782 and HCB/2022/0651). Patients signed an informed consent before surgery (agreeing the use of the low field-iMR and for the academic and scientific use of their anonymized data). The study complies with national legislation in the field of biomedical research, the protection of personal data (15/1999) and the standards of Good Clinical Practice, as well as with the Helsinki Declaration (1975 and 1983 revisions). Patient records were anonymized before analysis.

### Surgical technique and outcome measurements

Patients within the iMR and iUS cohorts were operated with conventional microsurgical techniques, including an ultrasonic aspirator and standard neuronavigation. In both groups, neurophysiologic monitoring was employed when the location of the lesion required motor cortical or subcortical mapping. Awake surgery was chosen for language mapping in suitable candidates. Functional criteria for stopping the resection remained unchanged across the duration of the whole study. To reduce the bias inherent to the variable degree of surgeons’ expertise with iUS operation, all interventions were performed by only two surgeons specialized in neurosurgical oncology.

The primary outcome was EoR, defined as Gross Total Resection (GTR) if at least 90% of the mass was removed; Near Total Resection (NTR) if at least 80% of the mass was removed; or Partial Resection (PR) if less than 80% of the mass was removed (14). In high grade gliomas and metastasis, the tumor mass corresponded to the contrast-enhancing lesion. In low grade gliomas, the lesion consisted of T2/FLAIR hyperintense infiltrative area. The secondary outcomes were the presence of surgical-related complications and the performance status at discharge (assessed by the Karnosfky Performance Status, KPS).

Postoperative complications included hemorrhage (epidural, subdural or intraparenchymal), wound infection, new neurological deficits, hydrocephalus, and venous thromboembolic disease. Other variables of interest were demographic (age and gender) and clinical variables (preoperative KPS), histopathological diagnosis, need of re-intervention within the first year, surgical duration, need for intensive care and total hospital length of stay.

### Economic analysis

Economic evaluation consisted of a cost-effectiveness analysis where the two intraoperative imaging techniques were compared, namely the low-field iMR with the iUS. The cost-effectiveness equation explored the incremental cost per unit of health gained with a given device. The effectiveness measures used were maintained or increased postoperative KPS and the EoR, expressed as a dichotomous variable, considering whether GTR was achieved or not achieved. The incremental effectiveness was expressed as the mean difference in the postoperative KPS and as the difference in the percentage of GTR achieved with each technique.

Health-related costs included health related variables [stay in the intensive care unit (days), hospital length of stay (days), type and number of radiological images performed before and after the intervention] and surgical-related variables, namely the operating time (in minutes), the use of prosthesis (dural substitutes, miniplates, hemostatic materials, etc.) and the use of neuronavigation system, the surgical pack and the intraoperative image device. The cost of the imaging device imputed to each patient was inferred as the cost per patient according to all the indications in which iMR or iUS are currently applied to, for the total lifespan of the device. Indications for iMR are intrinsic and extrinsic brain lesions, cavernomas, pituitary macroadenomas and epilepsy surgery, which comprises about 120 surgeries per year in our institution. Indications for iUS include intrinsic and extrinsic brain lesions, hydrocephalus and neurovascular interventions. These account for about 150 surgeries per year. The life cycle of both devices was set at 10 years. Although other health-related costs were described, they did not compute for the cost-effectiveness analysis (stay in the intensive care unit [days], hospital length of stay [days], type and number of radiological images performed before and after the intervention). Prices were extracted from our institution’s budget and cost of health credits. The same unitary prices were apply to both cohorts, even when they differ in eight years, so as to obtain comparable expenses (euro 2018). Therefore, no discount rates were applied. Costs were expressed as mean cost per patient.

The mean incremental cost and mean incremental effectiveness were calculated for each modality. The cost-effectiveness ratio (ICER) was defined as the ratio between the incremental cost and the incremental effectiveness of the two intervention alternatives, as follows:


$$ICER=\frac{Cost\;of\;iMR-Cost\;of\;iUS}{GTR\;with\;iMR-GTR\;with\;iUS}$$

The ICER values of the two intraoperative imaging variants were represented in a cost-utility plane. In this graphic, the north-east corner indicates a more expensive and more effective intervention, whereas the south-east corner indicated a less costly but more effective intervention. Finally, a graphical representation of the evolution of surgical times with the sequentially acquired experience of the surgical team was obtained for both techniques.

Calculations were performed using Microsoft Excel XPTM and SPSS (IBM version 23.0). The present analysis followed the Health Economic Evaluation Reporting Standards (CHEERS) guidelines for communicating economic evaluations of health interventions. No statistical tests were conducted as neither hypothesis testing, nor the level of statistical significance were relevant to our analysis.

### Brief literature review

To contextualize our results in terms of efficacy and efficiency, we ran a succinct literature review of the main trials and observational studies reporting the outcomes of the use of iMR and/or iUS for glioma resection. Concretely, we conducted a PubMed search with the words “intraoperative ultrasound” and/or “intraoperative magnetic resonance” and “glioma surgery”. Only studies reporting the rates of gross total resection were included. Small series or series older than 2005 were excluded. Results of the search were summarized in an informative table, along with our own current results, specifying the year of publication, the type of intraoperative imaging device used, the study design, the tumor type included, the sample size, the rates of gross tumor resection and the surgical duration (if available). No statistical analysis was performed to compare between the different studies.

## Results

A total of 67 patients were included for the analysis: 50 had an iMR assisted surgery and 17 had an iUS guided intervention. Patients in which iUS was only used to obtain a biopsy were excluded from the analysis. A detailed description of the iMR results and cost-effectiveness analysis has already been published by our group (13). The results regarding iUS and the comparison between the two techniques in terms of cost-effectiveness are original and had not been previously reported.

Both cohorts had a male preponderance, a mean age of 50-60 years and an overall good performance status preoperatively (median KPS 90). In both groups, the predominant tumor type was high grade glioma (62% in iMR vs 70% in iUS) (**Table 1**).

**Table 1:** Socio-demographic and clinical variables.

	iMR (n = 50)	iUS (n = 17)	p
Gender, female [n (%)]	20 (40)	5 (30)	0.160
Age [median (range)]	53 (21-82)	57 (47-74)	0.566
Preoperative KPS [median (range)]	90 (70-100)	90 (40-100)	0.232
Low grade glioma [n (%)]	19 (38)	5 (30)	0.156
High grade glioma [n (%)]	31 (62)	12 (70)	0.154

iMR, intraoperative magnetic resonance; iUS, intraoperative ultrasound; KPS, Karnofsky performance status.

### Clinical outcomes

Surgical resection of tumors assisted with iMR, compared to iUS, provided higher rates of complete resection and lower incidence of postoperative complications (**Table 2**). The potential benefit related to iMR is regarded as an observational trend, since no statistical comparison was performed, as this falls outside the objectives of this study. With iMR gross total resection was achieved in 70% of cases, with acceptable postoperative morbidity (median KPS 80, complication rate of 14% with 8% needing reintervention). Complications in the iMR group included three symptomatic hematomas, one CSF fistula, two cerebral focal ischemia and one new-onset epilepsy.

**Table 2:** Clinical outcomes and total cost per intervention type.

	iMR (n = 50)	iUS (n = 17)	Differential (iMR-iUS)
Gross total resection, n (%)	35 (70)	10 (60)	10
Complications, n (%)	7 (14)	4 (20)	- 6
Reintervention, n (%)	4 (8)	2 (11)	- 3
Postoperative KPS [median (range)]	80 (60-100)	80 (60-100)	0
Postoperative KPS equal or increased [n (%)]	37 (70)	11 (65)	5
**Total cost per intervention**	10,893	7,673	3,220
OR	5,162	3,186	1,976
ICU	472	326	146
Hospitalization	4,177	2,358	1,819
Diagnostic images	1,082	739	343

ICU, Intensive care unit; iMR, intraoperative magnetic resonance; iUS, intraoperative ultrasound; KPS, Karnofsky performance status; OR, operating room.

Conversely, with iUS complete resection was obtained in 60% of cases. Postoperative outcomes were similar in terms of performance status (mean KPS 80), yet morbidity was higher with iUS than with iMR. With iUS there was a 20% complication rate, which included two epidural hematomas, one surgical-cavity hematoma and one surgical-site infection. 11% of iUS-guided cases needed a reintervention due to surgical-related complications (**Table 2**).

### Health related costs

Mean cost per operation was higher if iMR had been used, ascending to € 5,162, compared to € 3,186 with the iUS. The number of patients requiring ICU and the mean length of hospital stay were also higher in the iMR setting (patients requiring ICU in iMR 34% vs 24% in iUS; mean LoS in hospital with iMR 10 days vs 6 days with iUS). Therefore, the total health-related costs for each intervention were higher with iMR assisted-surgery (€ 10,893) than with iUS guided-surgery (€7,673) (**Table 3**).

**Table 3:** Resources used and computed unit costs.

	iMR (n = 50)	iUS (n = 17)	Unit cost (€)
**OR**			
Time (min) [mean (SD)]	450 (70)	209 (47)	5
Surgical pack [% (n)]	100 (50)	100 (17)	1,150
Prosthesis [% (n)]	88 (44)	24 (4)	272
Navigation system [% (n)]	100 (50)	100 (17)	862
LF-iMR [% (n)]	100 (50)	0	833*
LF-iUS [% (n)]	0	100 (17)	67**
**ICU** [% (n)]	34 (17)	24 (4)	555
**Hospitalization** [mean LoS in days (SD)]	10 (5)	6 (2)	422
**Preoperative images** [mean (SD)]			
MR	1.35 (1.3)	1.18 (0.6)	170
PET	0.1 (0.3)	0.12 (0.3)	566
X-Ray	1.1 (1.2)	1.18 (0.5)	15
portable X-Ray	0.1 (0.5)	0	32
CT	0.4 (0.6)	0.47 (0.6)	72
**Postoperative images** [mean (SD)]			
MR	2.8 (1.65)	1.65 (0.7)	170
PET	0	0.06 (0.23)	566
SPECT	0.1 (0.3)	0	166
X-Ray	1.7 (3.5)	1.5 (0.8)	15
portable X-Ray	0.3 (0.9)	0.3 (0.2)	32
CT	0.7 (1.4)	1.12 (1.3)	72
**TOTAL UNIT COST** (€)	10,893	7,673	

ICU, Intensive care unit; iMR, intraoperative magnetic resonance; iUS, intraoperative ultrasound; LoS, Length of Stay; OR, operating room.

*Cost per intervention using iMR based on the life cycle (10 years) and the potential number of annual patients (n = 120) who benefit from the iMR device.

**Cost per intervention using iUS based on the life cycle (10 years) and the potential number of annual patients (n = 150) who benefit from the iUS device.

**Figure 1:**
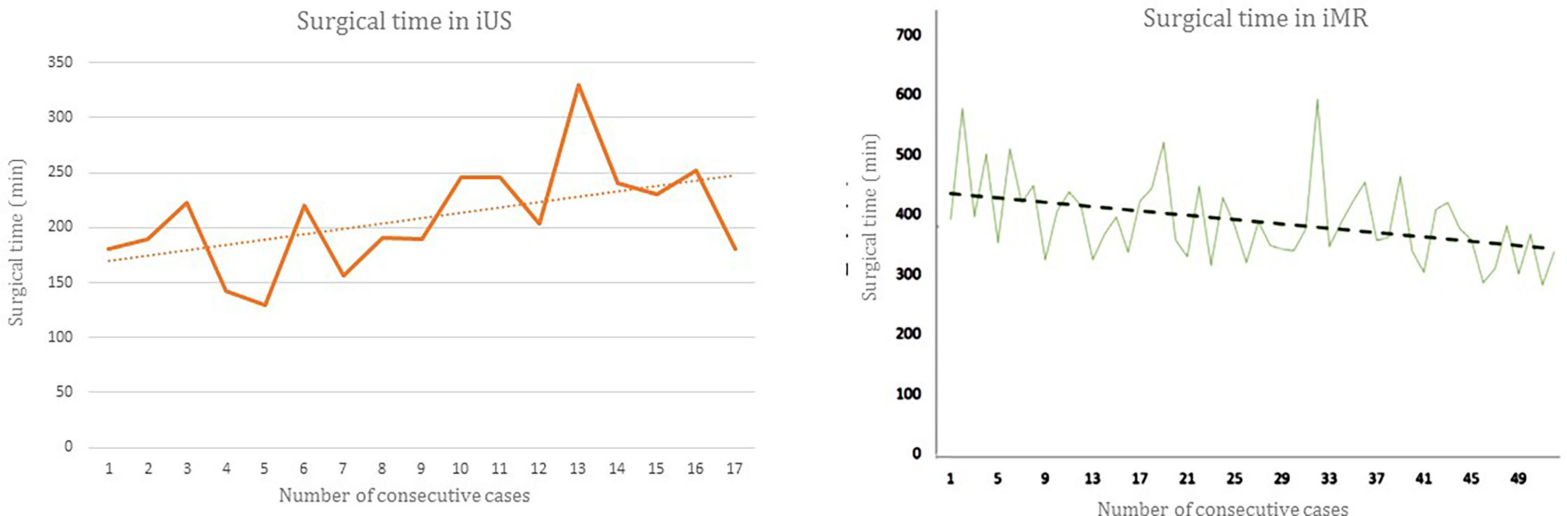
Surgical time per patient according to the intraoperative imaging device. The graphics illustrate the sequential evolution of surgical times required for each patient. The superimposed line demonstrates the trend of intraoperative duration as the experience increases with each imaging technology. *Left*, intraoperative ultrasound (iUS) and *Right*, intraoperative magnetic resonance (iMR). Reprinted with permission of García-García et al., 2020 (13).

Surgical duration was more than double when iMR was used than when iUS was chosen, with an average of 241 minutes more per intervention. Interestingly, the sequential evolution of surgical times was different for the two techniques: While iMR-surgery tended to become nimbler with time, a flat evolution of the iUS-surgery was observed (**Figure 1**).

### Cost-effectiveness analysis

The costs of iMR-assisted surgery were higher than with iUS (incremental cost per intervention of € 3,220). Meanwhile, the iMR seems more effective at achieving gross total removal of the tumor (mean percentage difference of 10 points). Still, postoperative performance status was similar with both techniques, but iMR showed slightly higher rates of equal or increased postoperative KPS (incremental benefit of 5 percentage points).

The results of the cost-effectiveness analysis (**Table 4**) reveal that, in terms of health-related costs, iMR seems cost-effective when compared to iUS in terms of complete tumor removal (ICER € 322 per GTR achievement) and postoperative performance status (ICER € 644 per KPS gained or maintained with iMR). These lines of results are maintained when only surgical-related costs are concerned, with and ICER of € 198 per GTR and an ICER of € 395 per KPS gained with iMR.

**Table 4:** Cost-effectiveness analysis.

	iMR	iUS	Difference	ICERSurgery-related	ICERHealth-related
Health-related cost (€)	10,893	7,673	3,220		
Surgical-related cost (€)	5,162	3,186	1,976		
Effectiveness measure (postoperative KPS equal or increased), %	70	65	5	395	644
Effectiveness measure (Gross total resection, % cases)	70	60	10	198	322

ICER, Incremental Cost-Effectiveness Ratio; iMR, intraoperative Magnetic Resonance; iUS, intraoperative UltraSound; KPS, Karnofsky Performance Status.

**Figure 2:**
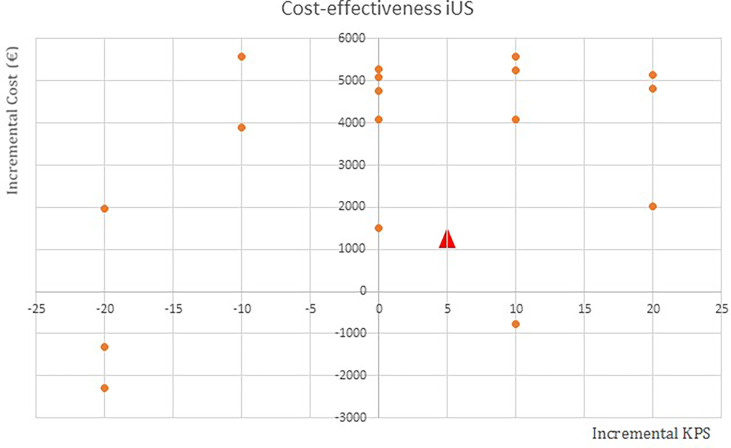
Cost-effectiveness plane of intraoperative ultrasound (iUS) compared to the intraoperative magnetic resonance imaging device. Each blue point represents a replicated case. The red triangle is the average of all the cases. X-axis, Effectiveness measure (KPS); Y-axis, Cost in euros.

In the cost-effectiveness plane representing the results of iUS compared to the iMR, nearly half of the replicates fall within the north-east corner, indicating a costlier and more effective intervention (**Figure 2**).

**Figure 3:**
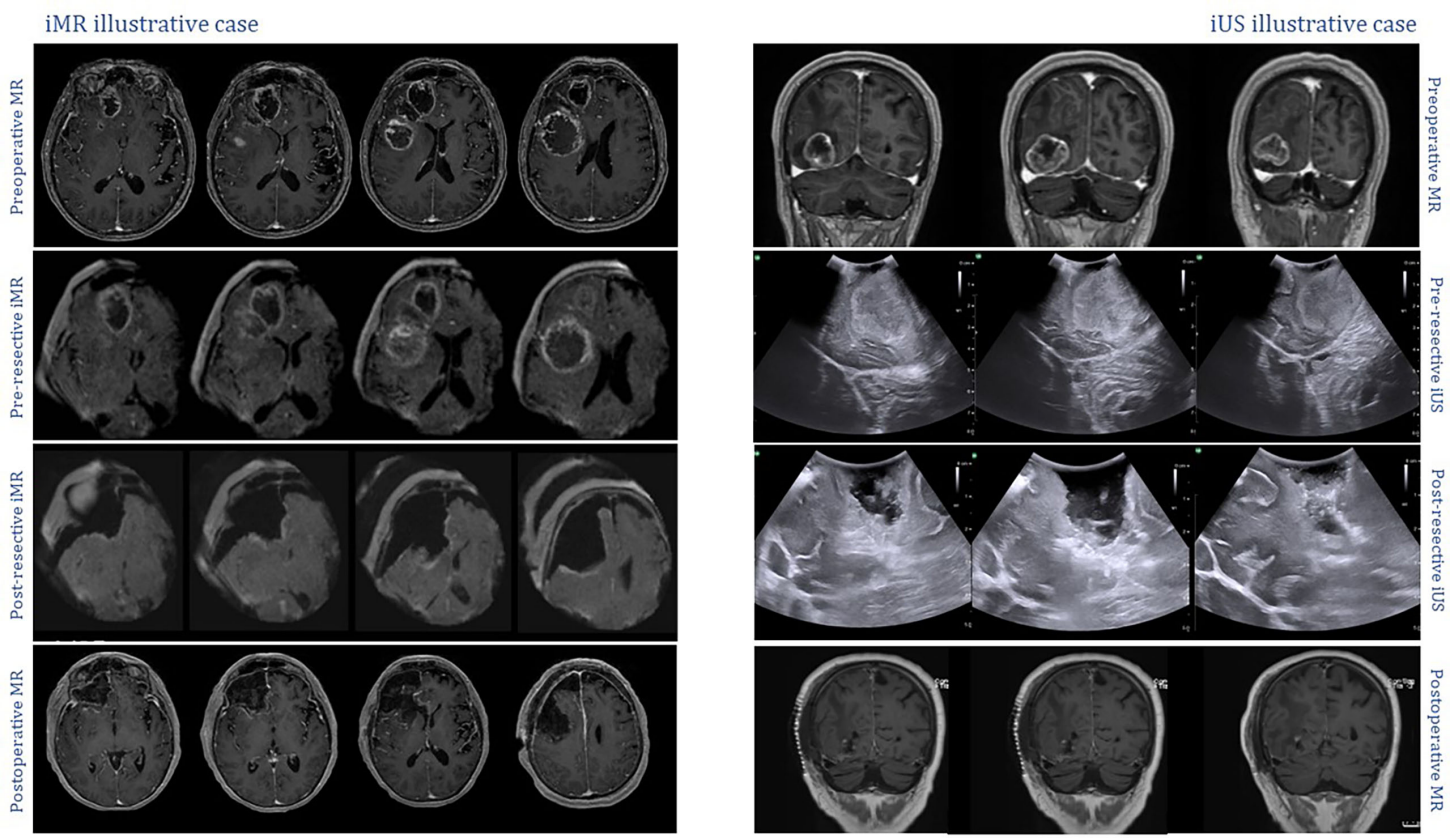
Intraoperative captures displaying examples of the imaging techniques undertaken during the study. *Left*, low-field iMR illustrative case. All images correspond to axial sections of T1 sequences after gadolinium administration. On the top row, preoperative MR study showing a right frontal lesion corresponding to a high-grade glioma; on the second row, initial iMR acquisition; on the third row, iMR control image obtained after resection, no residual disease can be seen around the surgical cavity; on the last row, postoperative MR confirming complete resection of the tumor. *Right*, iUS illustrative case. On the top row, preoperative MR T1+gadolinium coronal sections showing a right occipital high-grade lesion; on the second row, initial iUS exploration with a coronal view of the occipital lesion adjacent to the tentorium cerebelli; on the third row, iUS control exploration after surgical resection, with no apparent residual disease; on the last row, postoperative MR T1+gadolinium confirming complete resection of the tumor.

Examples of the intraoperative images used during the interventions can be seen in **Figure 3**.

## Discussion

This is an unprecedented but preliminary cost-effectiveness analysis comparing the two most commonly used intraoperative imaging devices in oncologic neurosurgery. Our results suggest that iMR, although being costlier and time-consuming, seems to be cost-effective comparing to iUS in terms of surgical resection rates, with an ICER of € 322 per GTR attained. A similar conclusion is obtained when only surgical-related costs are regarded, with and ICER of € 198 per GTR. On the other hand, when KPS was taken as the unit of health gain, slight differences were found among the two techniques; still, iMR seemed to be cost-effective to the iUS counterpart. Whether the apparent profitability of the iMR is worth the high initial investments required and the longer surgical duration times will depend on the willingness-to-pay threshold of each local healthcare system and the logistics policy of each institution.

Our economic analysis was performed under two different economic perspectives: one accounted only for the surgical-related costs, and the other one including all the total costs incurred during hospitalization. This strategy was intended to reduce the bias related to the differences in those costs not directly related to the intraoperative image of choice, such as systemic complications and length of stay. For instance, the higher rates of postoperative complications within the iUS-guided group might not be directly related to the imaging devise *per se* (potential selection bias). Meanwhile, a slightly poorer postoperative KPS within the iUS group might be the consequence of a more ambitious approach to resection, by which trying to achieve higher GTR rates there is collateral damage in the form of new neurological deficits (due to small vessel violation or grey/white matter disruption).

In both series, the intention of the surgeries was maximal safe resection. In cases of tumors located near eloquent cortical or subcortical structures, neurophysiological monitoring was performed. In both iUS and iMR cohorts there were cases in which resection was halted prematurely due to the proximity of functional areas. However, a plausible explanation for the difference (10%) in GTR between both techniques is a mismatched distribution in the functionally limiting tumor excisions. In fact, in our institution, iMR is used in well-selected candidates, in whom total tumor resection is pursued as a primary goal and in whom the total removal of the tumor seems feasible according to the preoperative planning. On the contrary, iUS is now used as a regular aid for tumor resection, even in cases where a complete removal was only sought up to some extent (potential selection bias).

Regarding surgical duration, the use of iMR increased operating times to near double those with iUS, a similar magnitude to what had been previously reported (15). Interestingly, duration seems to decrease with cumulative cases in the iMR device, but not so with the use of iUS. Perhaps, the workflow required for iMR involves the whole surgical team (surgeons, anesthesiologist and nurses), who progressively become more confident and agile with patient preparation and device mobilization. Conversely, iUS relies directly on the surgeon’s ability to acquire the desired projections and to correctly interpret the images. Consequently, the learning curve might be slower, and the number of interventions needed to decrease surgical times might exceed the contemplated 17 cases. Indeed, the interpretation of iUS results could become better with time and experience, and so would the resection rates.

Although the limited experience with iUS was also concerning at the beginning, the results obtained by our group are in line with previously reported series, **Table 5** summarizes the results so far reported about the efficacy of iUS guided glioma surgery (10, 16–19, 21 - 42). In this regard, a common obstacle for identifying residual tumor was the acoustic enhancement artifact, due to the liquefied surgical cavity. Some authors have suggested that serial iUS acquisitions during the resection may help differentiate between artifact and tumor at the end of the procedure (31); meanwhile, specific software is also becoming available (29). Another strategy is the use of sonographic contrast agents; even if the experience with these is limited, they seem to enhance the lesion borders compared with the standard B-mode iUS. Moreover, contrast-guided evaluation provides information about the tumor perfusion pattern, which could also facilitate the surgical procedure (32).

**Table 5:** Summary of the main trials and observational studies evaluating intraoperative ultrasound and magnetic resonance imaging for the resection of high and/or low-grade gliomas.

Author	Intraoperative image	Study type	Tumor type	Sample size(imaging)	Rates of gross total resection	Surgical duration (min), mean
Senft et al., 2011 (10)	iMR, ultra-low-field	Randomized trial	Glioma grade 4	24	96%	250
Kubben et al., 2014 (16)	iMR, ultra-low-field	Randomized trial	Glioma grade 4	6	50%	90-120 more than control
Wu et al., 2014 (17)	iMR, high-field	Randomized trial	Glioma grades 2-4	44	91% high-grade 82% low-grade	Not reported
Bai et al., 2015 (18)	iMR, high-field	Prospective controlled trial	Glioma grades 2-4. Language area	112	95%	Not reported
Incekara et al., 2015 (19)	iMR, high-field	Retrospective cohort study	Glioma grades 1-2.	29	93%	Not reported
Rorder et al., 2013 (20)	iMR, high-field	Retrospective cohort study	Glioma grade 4	27	74%	354
Schatlo et al., 2015 (21)	iMR, ultra-low-field	Retrospective cohort study	Glioma grade 4	55	45%	Not reported
Familiari et al., 2018 (22)	iMR, high-field	Retrospective cohort study	Glioma grades 3-4	64	67%	Not reported
Bassaganyas-Vancells et al. 2019 (23)	iMR, ultra-low-field	Retrospective cohort study	Glioma grades 3-4	58	72%	188
Fujii et al., 2022 (24)	iMR, low-field	Retrospective cohort study	Glioma grades 2-4	11	73%	466
Current study (Present study data)	iMR, low-field	Retrospective cohort study	Glioma grades 2-4	50	70%	450
Renner et al., 2005 (25)	iUS	Prospective series	Glioma grade 4 and metastasis	22	58%	Not reported
Moiyadi et al., 2013 (26)	iUS, navigated	Retrospective cohort study	Glioma grade 3-4	51	47%	264
Solheim et al., 2010 (27)	iUS	Retrospective cohort study	Glioma grade 4	142	37%	Not reported
Shetty et al., 2021 (28)	iUS, navigated	Retrospective series	Glioma grades 2-4	210	75%	Not reported
Current study (Present study data)	iUS	Retrospective cohort study	Glioma grade 2-4	17	60%	209

In studies where two groups are compared, the sample size refers to the group exposed to the intraoperative imaging.

Arguably, iMR provides better image resolution, tissue differentiation and wider field of view. These intrinsic characteristics are conceivably responsible for the greater tumor resection rates (33). Notwithstanding, iUS is a currently evolving field, with advances like elastography (34), the use of contrast agents (32), integration with preoperative MR navigation (35, 36), along with the increased experience in the neurosurgical ground. Thus, iUS might soon proof effective to increase resection rates to as close as those obtained with iMR. In such a case, iUS would become more cost-effective and certainly more attainable for the general public, given the lower initial investment required.

Possibly, iMR and iUS are complementary aids in surgical neuroncology. During the resection, iUS provides real-time information while the surgeon is advancing towards the tumor limits, informing about the distance to relevant landmarks, such are the ventricles or blood vessels, and correcting neuronavigation inaccuracy due to brain shift and deformation (28, 37). Yet, at the end of the resection, it is the iMR that would reliably corroborate whether residual tumor has been left (38).

## Limitations

Limitations of the present study include the time lapse between the collection of iMR series and iUS series of patients and the heterogeneity of both cohorts. Even when the general management of oncologic patients has not significantly changed over the last decade, advances in neuronavigation and improved experience in neurophysiologic mapping might have acted as bias when comparing the primary and secondary clinical outcomes between the two series. Probably, another source cofounding is the use of gross total resection as the unit of health gain; even when this parameter is of great clinical relevance, its achievement is not only related to the ability of detecting residual tumor. In fact, the surgical aim in this study was a maximal safe resection, and thus safety (e.g., neurophysiological alert, closeness to critical areas like the ventricles, the main vessels, the brainstem, etc.) might preclude the cautious surgeon from total resection. To add to this variability, it should be noted that provided the iUS is a highly operator-dependent technique, particularly compared to iMR, the reliability of the results regarding surgical duration and quality of resection are strictly linked to the surgeon’s experience and expertise.

Finally, certain aspects of the study design should be addressed. The limited sample size, particularly in the iUS group, could be a source of deviation of the global results; however, this study was not intended to prove the superiority of one intraoperative technique over the other. Conversely, this economic evaluation is meant to help in health-related decision making during the set-up of novel operative armamentarium. In fact, the decision process underlying a cost-effectiveness analysis should be based only on the mean net benefits of each intervention irrespective of whether the difference between them is statistically significant (39, 40). Certainly, cost-effectiveness studies are typically performed within or after efficacy trials; nonetheless, no randomized trials are currently available comparing iMR and iUS in neurosurgical oncology. On the other hand, economic evaluation is not typically concerned with hypothesis testing, is rather more an estimation, and thus could still provide useful information even when under-powered.

## Conclusion

In intracranial oncological procedures, iMR and iUS seem to afford similar results in terms of extent of resection and postoperative performance status; still, the outcomes slightly favor iMR although at a higher relative cost and with longer surgical times. Surgical duration decreases with cumulative experience with iMR, but not so much with the use of iUS, reflecting the obvious differences in the intraoperative workflows between both techniques; while iMR involves the whole surgical team becoming familiarized with patient preparation and device mobilization, iUS relies directly on the surgeon’s ability to simultaneously acquire and interpret the examination images. Possibly, iMR and iUS are complementary aids in neurosurgical oncology: Whilst iUS assists the surgeon with real-time captures while advancing towards the tumor limits, informing about the distance to relevant landmarks and correcting neuronavigation inaccuracy due to brain shift; at the end of the resection, it is the iMR that reliably corroborates whether residual tumor remains.

## Data availability statement

The raw data supporting the conclusions of this article will be made available by the authors, without undue reservation.

## Ethics statement

The studies involving human participants were reviewed and approved by Comité de Etica de Investigación Clínica, Hospital Clínic de Barcelona, Spain. Written informed consent for participation was not required for this study in accordance with the national legislation and the institutional requirements.

## Author contributions

AM, AS, and JG designed and led the present study. JG, AS, and PR developed the theory and performed the computations. AM, AF, and AR collected the clinical and economic data. AM, AS, SG-O, and JG performed the analytical methods and interpreted them. AM drafted the manuscript. JG, AS, and PR critically revised the manuscript. JE gave institutional, material, and logistic support. All authors contributed to the article and approved the submitted version.

## Conflict of interest

The authors declare that the research was conducted in the absence of any commercial or financial relationships that could be construed as a potential conflict of interest.

## Publisher’s note

All claims expressed in this article are solely those of the authors and do not necessarily represent those of their affiliated organizations, or those of the publisher, the editors and the reviewers. Any product that may be evaluated in this article, or claim that may be made by its manufacturer, is not guaranteed or endorsed by the publisher.
